# Editorial: Circadian rhythms and aging

**DOI:** 10.3389/fnins.2026.1910344

**Published:** 2026-07-14

**Authors:** Javier Alamilla, Eloy Gerardo Moreno-Galindo, Jennifer C. Tudor, Charles N. Allen

**Affiliations:** 1 Centro Universitario de Investigaciones Biomédicas (CUIB), Universidad de Colima, Colima, Mexico; 2 Secretaría de Ciencia, Humanidades, Tecnología e Innovación (SECIHTI), Universidad de Colima, Colima, Mexico; 3 Department of Biology, Saint Joseph's University, Philadelphia, PA, United States; 4 Oregon Institute for Occupational Health Sciences, Oregon Health and Science University, Portland, OR, United States; 5 Department of Behavioral Neuroscience, Oregon Health and Science University, Portland, OR, United States

**Keywords:** development, health aging, older population, rhythmicity, synchronization to light

In most living organisms, circadian rhythms are physiological and behavioral fluctuations with a periodicity of approximately 24 h, thought to have been ancestrally induced by the light-dark cycle of the day-night succession. These rhythms provide significant biological advantages by preparing organisms for day-night cycles and associated activities. Human society has rigid schedules for work, school, and social activities. These schedules frequently require people to work and be active when their endogenous circadian clock signals that it is time to sleep. Synchronized circadian rhythmicity is necessary for overall health, and circadian disruption leads to premature death and notable changes in the brain, metabolism, and behavior.

Advances in healthcare and living conditions have significantly increased life expectancy; consequently, the global aging population is placing unprecedented demands on healthcare services and medical research. Aging alters circadian rhythms, both behaviorally (sleep disturbances) and physiologically, by decreasing their strength (lower amplitude) and duration (reduced period), weakening their synchronization with environmental light cues, and almost certainly impacting the health of the elderly (Farajnia et al., 2014).

The eight manuscripts in this Research Topic explore the interrelated domains of aging, sleep, circadian rhythms, and neurological or physiological function. The four review articles synthesize current knowledge on circadian biology, sleep regulation, and therapeutic strategies. This Research Topic also includes four original research papers that provide experimental and observational evidence that deepens understanding of these processes. Together, they form a coherent body of work centered on aging-related changes in sleep and circadian systems, as well as potential interventions.

Among the review papers, Drǎgoi et al. highlight the importance of melatonin circadian regulation and propose several strategies to improve its rhythmicity. The review introduces the concept of *chrononutrition*, emphasizing that the timing of food intake and consumption of melatonin-rich foods can enhance circadian alignment and mitigate age-related disorders. The authors argue for combined chronobiological and nutritional interventions, including melatonin supplementation and lifestyle alignment with light–dark cycles, as promising approaches to extend health span. Qu et al. used a structured scoping review methodology to conclude that both multi- and single-sensory stimulation therapies (auditory, visual, olfactory, and tactile) can positively impact sleep quality, including sleep latency, total sleep time, and subjective sleep experience. Liu et al. emphasize the role of chronotype in regulating cognitive function through circadian modulation, neuroinflammatory processes, and metabolic homeostasis. Their review highlights how variations in chronotype contribute to perioperative disruptions to circadian rhythms—such as reduced slow-wave sleep and circadian misalignment and may contribute to postoperative cognitive dysfunction (POCD). The review underscores the importance of considering chronotype in clinical care and suggests that targeted circadian interventions could reduce postoperative cognitive complications. Finally, Su and Xiang underscore the importance of aligning physical activity with chronotype to enhance circadian synchronization. They present the concept of “chrono-exercise,” timing physical activity in alignment with circadian rhythms, as a proposed strategy to optimize muscle repair and delay functional decline in older adults.

Original research was reported by Zhào et al. that circadian rhythm disruption is associated with increased postural sway and a higher risk of falls, particularly when visual input is limited. These findings suggest that circadian misalignment may contribute to fall risk in aging populations, highlighting the importance of maintaining circadian integrity for physical stability. In turn, Feeney et al. found that overexpression of calcium/calmodulin-dependent protein kinase IV (CaMKIV), a protein closely linked to memory consolidation, enhanced both the non-rapid eye movement (NREM) and rapid eye movement (REM) sleep in aged male mice, but not in young adult or female counterparts. The findings indicate that CaMKIV plays a role in age- and sex-dependent regulation of sleep architecture, potentially through CREB signaling pathways. This research describes a putative molecular mechanism underlying aging-induced changes in sleep regulation. Fu et al. observed that both young and older adults exhibit changes in gray matter, cortical thickness, and cortical surface area across different brain regions following acute sleep deprivation. The findings reveal distinct patterns: young adults exhibit localized decreases and increases in cortical regions, whereas older adults show more widespread increases in cortical measures. These results suggest that partial sleep deprivation affects brain structure differently across the lifespan, highlighting age-specific vulnerability and adaptive mechanisms. Finally, Li et al. used computational and experimental methods to investigate the mechanisms of the Bushen Anzhi recipe (Chinese herbal medicine) in the treatment of aging-related insomnia. Their findings reveal that treated animals exhibited increased total sleep time and slow-wave sleep, as well as improved memory and exploratory behavior. These effects were associated with the regulation of key target genes, including H1F1A, PTGS2, and GSK3B, suggesting a potential role for the *Wnt* signaling pathway.

Collectively, the studies included in this Research Topic highlight the central role of circadian rhythms in the aging process and their broad impact on sleep, cognition, metabolism, and overall health. Importantly, they disclose that age-related circadian disruption is not irreversible but rather adjustable. Future research should prioritize integrative and personalized approaches that combine chronobiology, lifestyle interventions, and targeted therapies to preserve circadian integrity and promote healthy aging.
